# Editorial: Translational research in hereditary spastic paraplegias: filling the diagnosis gap and therapeutic perspectives

**DOI:** 10.3389/fnins.2025.1595717

**Published:** 2025-04-10

**Authors:** Andrea Martinuzzi, Craig Blackstone, Giovanni Stevanin

**Affiliations:** ^1^Department of Public Health and Pediatrics, University of Turin, Turin, Italy; ^2^Department of Neurology, Massachusetts General Hospital, Boston, MA, United States; ^3^INCIA, EPHE, CNRS, University of Bordeaux, Bordeaux, France

**Keywords:** hereditary spastic paraplegia (HSP), outcome measures, biomarkers, treatment, therapeutic targets

Several years have passed since *Frontiers in Neuroscience* published a special topic issue in 2021 focusing on the crossroads of molecular pathways and clinical options for the hereditary spastic paraplegias (HSPs) (Martinuzzi et al., 2021). We now present a novel special topic clearly showing how we moved forward from that crossroad toward research and experiences that prefigure a transition to a fully translational phase for HSPs.

## Improving knowledge on disease progression and identifying biomarkers

The HSPs are a large, clinically and genetically diverse group of inherited neurologic disorders united by a common feature of lower extremity spasticity and gait impairment due to a length dependent axonopathy of corticospinal motor neurons. Despite advances in genetic diagnosis, HSPs remain largely untreatable, with current management limited to symptomatic relief via physiotherapy, muscle relaxants, and environment adaptation. A major barrier to developing effective treatments has been a lack of reliable biomarkers and clinical outcome measures that can accurately and sensitivity track disease progression and evaluate therapeutic response. Given the slow and variable progression of HSPs, long-term studies incorporating both clinical performance measures and neuroimaging or serum biomarkers are essential for identifying disease-modifying targets and designing clinical trials.

In this issue, two studies presented distinct but complementary aspects of HSP pathology assessment. The study by Cubillos Arcila et al. aimed to characterize the long-term progression of hereditary spastic paraplegias (HSPs) through a 4.5-year follow-up of patients with confirmed genetic diagnoses (26 HSP patients with SPG4, SPG7, SPG5, or SPG3A). By tracking clinician-reported (including the spastic paraplegia rating scale SPRS) and performance-based (walking tests, locomotor rehabilitation index, Timed Up & Go test) outcomes, the researchers sought to identify sensitive measures of disease progression that could inform clinical trial design for future treatments. Performance-based measures were more sensitive to change than clinician-reported measures, and sample size calculations suggested that the Timed Up & Go test requires the smallest patient cohort to detect disease improvement.

The objectives of the second study by Montanaro et al., were to explore brain metabolic changes in HSP patients using magnetic resonance spectroscopy (MRS), a non-invasive neuroimaging technique that quantifies key metabolites related to neuronal integrity, inflammation, and energy metabolism. The goal was to determine whether MRS biomarkers could differentiate HSP patients (*n* = 46) from healthy controls (*n* = 46) as well as to track disease progression over time. Worsening of the SPRS score correlated with quantifiable metabolic changes. Among these changes, progressive myo-inositol/creatine levels in the pre-frontal cortex may indicate chronic neuroinflammation, alterations in oligodendrocyte-mediated myelination and impaired motor protein intracellular cargo transport, as suggested previously (Blackstone, 2012), together with neuronal loss or dysfunction observed by reduced levels of *N*-acetylaspartate/creatine. SPG4 patients also had lower choline/creatine levels, opening the way for MRS-based HSP classification in the future, after enrollment of more SPG patients. Applying an inference tree methodology MRS differentiated HSP subjects and controls with an overall accuracy of 73.9%, a sensitivity of 87.0%, and a specificity of 60.9%.

By integrating functional and biochemical assessments, these 2 studies pave the way for more precise disease monitoring and personalized treatment approaches in HSP research. Future studies should explore targeted therapies with MRS and gait performance measures as primary outcome metrics.

## Finding therapeutic targets

To better understand the physiopathology of HSPs, investigating the functions of the genes involved is crucial. Despite the large number of implicated genes (>80), the affected cellular processes converge on a few key functions, such as organelle shaping, intracellular trafficking, mitochondrial functions, lipid metabolism, and development/myelination (Van de Vondel et al., 2024). Interconnections among these functions suggest that common mechanisms may underlie various forms of HSP, potentially unveiling shared therapeutic targets (Toupenet Marchesi et al., 2021). Studying disease-relevant models can also provide critical insights into these mechanisms, as demonstrated in two studies in this issue focusing on two rare neurodegenerative diseases: autosomal recessive spastic ataxia of Charlevoix-Saguenay (ARSACS) and hereditary spastic paraplegia type 11 (SPG11). Both diseases are marked by progressive neurodegeneration, with ARSACS primarily involving spastic ataxia and cerebellar dysfunction due to mutations in the *SACS* gene, while SPG11 presents spastic paraplegia, cognitive impairment, and psychological abnormalities due to mutations in the *SPG11* gene.

The *SACS* gene encodes sacsin, a multifunctional protein crucial for mitochondrial function, protein quality control, cytoskeletal organization, and intracellular trafficking (Romano et al., 2013; Morani et al., 2019). In ARSACS, restoring calcium (Ca^2^^+^) influx in neurons has previously been shown to improve motor symptoms, arrest Purkinje cell degeneration, and reduce neuroinflammation in the cerebellum of *Sacs* knockout mice (Del Bondio et al., 2023). The present study by Galatolo et al. used unbiased proteomic and targeted lipidomic analyses to explore ARSACS pathophysiology, identifying disrupted lipid metabolism, impaired Ca^2^^+^ signaling, and oxidative stress as key contributors to disease progression. Abnormal intracellular Ca^2^^+^ homeostasis, a critical factor for cellular signaling and mitochondrial function, was confirmed in ARSACS fibroblast cells. The authors suggest that elevated ceramide levels and reduced diacylglycerols are linked to mitochondrial dysfunction, highlighting lipid and Ca^2^^+^ dysregulation as central to ARSACS pathology. These findings suggest potential biomarkers and therapeutic targets for the disease, emphasizing the importance of maintaining lipid and Ca^2^^+^ homeostasis in neurodegeneration.

The *SPG11* gene encodes spatacsin, a protein involved in lysosome recycling. In SPG11, earlier studies have revealed significant lipid metabolism impairments, including the accumulation of cholesterol and gangliosides in lysosomes of SPG11 cells (Boutry et al., 2018, 2019), alongside disrupted Ca^2^^+^ homeostasis. Transcriptomic analyses of knockout models have also demonstrated altered inflammatory responses (Toupenet Marchesi et al., 2025), while CD8 T lymphocytes have been suggested to exacerbate axonal damage, with targeting the adaptive immune response improving motor phenotypes in the *Spg11* knock-out mouse model (Hörner et al.). Building on these findings, the current study by Hörner et al. investigated the role of adaptive immune-mediated neuroinflammation in SPG11 by genetically inactivating the immune response (via *Rag1* knockout) and testing the immunomodulators fingolimod (FTY720) and teriflunomide. Behavioral analyses of SPG11 mouse models revealed profound social behavior deficits, hyperactivity, and anxiety-like traits, mirroring some patient symptoms. Both genetic inactivation of the adaptive immune system and treatment with fingolimod or teriflunomide, drugs currently approved for multiple sclerosis, successfully mitigated behavioral abnormalities. Additionally, these interventions preserved cerebellar Purkinje cells, essential for motor coordination and social behavior, identifying neuroinflammation as a viable therapeutic target for SPG11.

Taken together, these two studies emphasize the critical roles of lipid metabolism, calcium signaling, and neuroinflammation in the pathophysiology of ARSACS and SPG11. By highlighting shared mechanisms underlying neurodegeneration, they provide a foundation for developing common therapeutic strategies for these rare disorders.

## Non-disease specific therapeutic options

While awaiting an effective therapy, current treatment options for HSPs rely on rehabilitation strategies, and these are the focus of the papers from Di Ludovico et al. and Boccuni et al. The first presents the state of the art in physical therapy (PT) approaches to HSP with a narrative review that concludes with a call for rigorously testing protocols where various techniques are integrated; the second presents an innovative protocol for doing just that. Di Ludovico et al. gather works published over the last two decades on PT treatments for HSP. Papers covering electrical stimulation (11 patients in a sham controlled cross-over study), repetitive magnetic stimulation (7 HSP subjects in a sham-controlled study), hydrotherapy (9 patients), physical training (one case), robot assisted training (13 patients), and balance training with virtual reality (protocol published, no results) are presented and discussed. While each approach yielded some positive results in isolated areas, no intervention was able to show efficacy on all the most relevant dimensions (tone, strength, posture, and balance maintenance, gait, quality of life). Importantly, the protocol described in the paper by Boccuni et al. responds to the call, presenting in detail the protocol of a multidimensional treatment addressing flexibility (tone), resistance (strength), movement execution (gait and balance), and aerobic capacity (fatigue) intended for 20 HSP adult patients. The treatment is organized along 5 to 10 weeks, with sessions incorporating all four targets with appropriate methodologies. Addressing in an integrated way the multifaceted functional impairments associated with HSP may overcome the sometime contradictory results thus far reported with mono-dimensional interventions. This protocol, already IRB-approved, is a solid initiative to address the complex problems leading to disability in HSP using a strong, evidence-based approach.

## Conclusion

The HSP field is really moving from an exploratory phase of identifying new genes and understanding pathophysiology of HSP, with much weight on the bench work of basic research, to a proactive, translational phase where knowledge is directed toward the identification of appropriate outcome measures and biomarkers, innovative therapies, and optimized interventions. The amazing speed of new data acquisitions through research is paying dividends, providing improved trial readiness and new hope for patients with HSP.
